# Autoimmune Pathology in Myasthenia Gravis Disease Subtypes Is Governed by Divergent Mechanisms of Immunopathology

**DOI:** 10.3389/fimmu.2020.00776

**Published:** 2020-05-27

**Authors:** Miriam L. Fichtner, Ruoyi Jiang, Aoibh Bourke, Richard J. Nowak, Kevin C. O’Connor

**Affiliations:** ^1^Department of Neurology, School of Medicine, Yale University, New Haven, CT, United States; ^2^Department of Immunobiology, School of Medicine, Yale University, New Haven, CT, United States; ^3^Trinity Hall, University of Cambridge, Cambridge, United Kingdom

**Keywords:** myasthenia gravis, B cells, B lymphocytes, autoimmunity, immunopathology, autoantibodies, AChR, MuSK

## Abstract

Myasthenia gravis (MG) is a prototypical autoantibody mediated disease. The autoantibodies in MG target structures within the neuromuscular junction (NMJ), thus affecting neuromuscular transmission. The major disease subtypes of autoimmune MG are defined by their antigenic target. The most common target of pathogenic autoantibodies in MG is the nicotinic acetylcholine receptor (AChR), followed by muscle-specific kinase (MuSK) and lipoprotein receptor-related protein 4 (LRP4). MG patients present with similar symptoms independent of the underlying subtype of disease, while the immunopathology is remarkably distinct. Here we highlight these distinct immune mechanisms that describe both the B cell- and autoantibody-mediated pathogenesis by comparing AChR and MuSK MG subtypes. In our discussion of the AChR subtype, we focus on the role of long-lived plasma cells in the production of pathogenic autoantibodies, the IgG1 subclass mediated pathology, and contributions of complement. The similarities underlying the immunopathology of AChR MG and neuromyelitis optica (NMO) are highlighted. In contrast, MuSK MG is caused by autoantibody production by short-lived plasmablasts. MuSK MG autoantibodies are mainly of the IgG4 subclass which can undergo Fab-arm exchange (FAE), a process unique to this subclass. In FAE IgG4, molecules can dissociate into two halves and recombine with other half IgG4 molecules resulting in bispecific antibodies. Similarities between MuSK MG and other IgG4-mediated autoimmune diseases, including pemphigus vulgaris (PV) and chronic inflammatory demyelinating polyneuropathy (CIDP), are highlighted. Finally, the immunological distinctions are emphasized through presentation of biological therapeutics that provide clinical benefit depending on the MG disease subtype.

## Introduction

Myasthenia gravis (MG) is an autoimmune disorder affecting neuromuscular transmission. MG patients suffer from muscle weakness and increased muscle fatigability due to diminished neuromuscular signaling (1, 2). The impairment in autoimmune MG is caused by autoantibodies that target components of the neuromuscular junction (NMJ) (1). The different subtypes of MG are defined by the antigen specificity of the autoantibody (2, 3). The most common subtype of autoantibody-mediated MG (approximately 85% of patients) is characterized by autoantibodies against the nicotinic acetylcholine receptor (AChR) (2). In the remaining 15% of patients, autoantibodies targeting muscle-specific kinase (MuSK) (4) or lipoprotein receptor-related protein 4 (LRP4) (5, 6) can be found. Another small fraction of patients does not have detectable circulating autoantibodies to known targets. Accordingly, these patients are diagnosed as having seronegative MG (SNMG).

Numerous *in vitro* approaches have substantiated that autoantibodies against AChR and MuSK in MG are pathogenic (3, 7–11). Their pathogenic capacity has been further demonstrated through passive transfer of patient-derived serum or immunoglobulin (12), maternal-fetal autoantibody transmission (13, 14), and neonatal transfer (15, 16), all of which reproduce MG symptoms. The direct role of autoantibodies in the pathology of MG places it in a rare category of autoimmune diseases caused by autoantibodies with well-established pathogenic affects. Accordingly, MG serves as an archetype for B cell-mediated autoimmune disorders.

Although MG patients with different subtypes share similar disease presentations, the underlying immunopathology of several subtypes are remarkably distinct, contradicting the uniformity in the disease phenotype. MG subtypes share features broadly associated with MG, which can be elicited by clinical examination (17, 18). However, without the results of autoantibody testing in-hand, it is not possible to uniformly assess the subtype through clinical examination alone. Thus, autoantibody testing is necessary for establishing the MG subtype. AChR and MuSK MG, in particular, highlight the distinct immunopathology of the subtypes. The immunopathology of AChR MG is characterized by IgG subclasses (IgG1, IgG2, and IgG3) with effector functions that can mediate tissue damage at the NMJ. AChR-specific autoantibodies are thought to originate from long-lived plasma cells. Conversely, MuSK MG is largely caused by autoantibodies with an IgG subclass (IgG4) that mediates pathology through the direct disruption of AChR signaling by interfering with NMJ protein-protein interactions. Short-lived plasmablasts are thought to be the source of these autoantibodies (19). These stark differences in immunopathology have been elucidated through laboratory-based studies and reinforced through both successful and failed outcomes in the testing of biological therapeutics. A deeper understanding of the mechanisms underlying the differences in immunopathology is highly important for both the patient and clinician – the accurate determination of autoantibody-related subtype has important consequences for care. Treatments that are anticipated to work well in one subtype may not have a biological basis for use in the other subtype(s).

In this review, we focus on the most common subtypes of MG. Rare congenital, presynaptic autoimmune, and thymoma-associated subtypes of MG do exist, but they are not discussed here and are reviewed elsewhere (20–22). The LRP4 and SNMG subtypes are presented, but given the limited information about the underlying immunobiology, they are not emphasized throughout. Rather, the immunobiology underlying the AChR and MuSK subtypes of MG are highlighted. Particular attention is given to AChR and MuSK autoantibody characteristics, B cell subsets, mechanisms of immunopathology, and the effects of treatment with biological agents. Insight is drawn from laboratory-based research using human specimens, clinical trial outcomes, and parallels to other autoimmune diseases.

## Immunopathology of AChR Myasthenia Gravis

### Characterization of B Cells in AChR Myasthenia Gravis

AChR MG can be divided into subtypes that are defined, in part, by age of onset and gender (23, 24). Patients who develop the disease before the age of 40–50 are often women. This subset is termed early-onset (EOMG), while those developing disease after the age of 40–50 fall into the late-onset LOMG category and are more often men. Patients in the EOMG category generally have conspicuous morphological changes of their thymus. This is primarily characterized by follicular hyperplasia and the presence of B cells and antibody secreting cells that organize into structures that share the characteristics of germinal centers (25–28). These structures are observed in approximately 70% of EOMG patients (29). There is a considerable amount of data that points to a major role in both the initiation and sustained production of AChR autoantibodies by B cells in the hyperplastic thymus. It was found that AChR-specific IgG (30) is present in the thymus along with activated B cells (31). A fraction of these activated B cells produces AChR-specific autoantibodies (32–34). The *in vitro* production of AChR autoantibodies by thymus-resident B cells can be spontaneous or driven by mitogens. Cells that spontaneously produce autoantibodies are most likely resident plasmablasts or plasma cells – both of which are known to occupy thymus tissue (32, 33, 35, 36). Thymic B cells requiring *in vitro* stimulation to produce autoantibodies are likely memory B cell populations, which require additional signals in order to differentiate to an antibody-secreting cell (ASC) phenotype (37, 38). Further confirmation that the thymus contributes to AChR autoantibody production was achieved through transplantation of thymus tissue from AChR MG patients into immunodeficient mice. AChR-specific autoantibodies were observed to deposit at the NMJ in these mice, demonstrating that AChR MG thymic tissue is sufficient for the production of AChR-specific antibodies and can cause muscle weakness in this rodent model (39). Sequencing of thymic B cell receptor repertoires identified clonal expansions of B cells in the thymus, although it has not been established if these expanded clones are specific for AChR (40, 41). The isolation of AChR-specific mAbs has provided additional details regarding the nature of the B cell repertoire producing them. Given that autoantibody-producing B cells are enriched in the thymus of many AChR MG patients (32, 36), several studies have used thymus tissue to isolate AChR-specific B cells. Sequencing of the antibody-variable regions afforded the characterization of these autoreactive B cells. It was demonstrated that B cells expressing AChR autoantibodies are clonally heterogeneous, class switched, and have accumulated somatic hypermutations (41–43), all of which are properties of antigen-driven maturation. At this time, a limited set of human AChR-specific B cells have been isolated. Studies using newer single cell technologies are certain to provide larger sets, so that the B cell receptor repertoire characteristics of these autoantibody-producing cells can be better understood.

In both LOMG and EOMG patients, autoantibody-producing B cells can occupy other tissue compartments in addition to the thymus. Using *in vitro* cell culture approaches, it has been demonstrated that B cells expressing AChR autoantibodies exist within the circulation, lymph nodes, and in the bone marrow (44–48). Other studies have identified autoantibody-producing B cells in the circulation through production of recombinant human monoclonal AChR autoantibodies (mAb) from these cells (49).

### Properties of AChR-Specific Autoantibodies

The first recombinant AChR-specific autoantibodies were cloned from phage display libraries isolated from thymocyte-associated immunoglobulin sequences (31, 43, 50). Several additional AChR-specific human-derived mAbs have been produced using a number of different approaches, including single-cell technology (49). These recombinant human-derived mAbs emulate the properties of AChR autoantibodies found in the serum: They compete for binding to regions of the AChR recognized by serum-derived autoantibodies (50), and they possess pathogenic properties demonstrated through passive transfer of MG (49). These mAbs, coupled with investigations using human serum-derived AChR autoantibodies, have provided a clear illustration of the three AChR autoantibody pathogenic mechanisms. The first pathogenic mechanism is the inhibition of acetylcholine binding to the AChR. These autoantibodies can block this interaction by either binding to the same site on the AChR, or in proximity to the binding site, which results in inhibition of acetylcholine-dependent signaling at the NMJ (51–53). The second mechanism is termed antigen modulation, which results in internalization of the AChR following autoantibody-mediated crosslinking. Antibodies are structured as dimeric molecules that have two identical heavy and light chain pairs with two antigen binding sites and a constant region that determines the effector function. Monovalent antigen-binding fragments (Fabs), which are derived from whole antibodies, have one single antigen binding site. These Fabs have been shown to lack the ability to crosslink the AChR, while whole antibodies can crosslink the AChR through bivalent binding with two binding sites. Subsequent to the receptor crosslinking, there is internalization of the AChR, which diminishes the number of receptors at the NMJ (54, 55). Finally, the third pathogenic mechanism involves the immunoglobulin effector functions of the AChR autoantibodies. The effector functions of IgG1 and IgG3 are key properties of their pathogenic capacity. Among their principle effector functions is the ability to initiate the complement cascade. AChR autoantibodies are predominantly of the IgG1 or IgG3 subclass and effectively activate complement, leading to the formation of the membrane attack complex and consequent tissue damage at the NMJ (56–59). Early studies demonstrated that complement-mediated damage to the postsynaptic NMJ results in reduction of the postsynaptic junctional folds, elimination of AChR from the membrane, and an increase in synaptic distance (59, 60). It is unmistakable that complement plays a key role in AChR MG pathology, given the successful treatment of patients (61) with complement inhibitors (discussed below).

### Similarities Between Neuromyelitis Optica and AChR Myasthenia Gravis

Parallels between AChR MG and autoimmune neuromyelitis optica (NMO) suggest that additional studies on the role of complement in AChR MG are warranted. Like AChR MG, NMO is mediated by pathogenic autoantibodies, primarily of the IgG1 and IgG3 subclasses, that include complement activation among the mechanisms of autoimmune pathology (62). Studies of complement in NMO have led to a detailed understanding of pathogenic mechanisms, which may take place in AChR MG as well. In NMO, aquaporin-4 (AQP4)-IgGs targeting a distinct epitope in an extracellular loop, regardless of affinity, enhanced complement dependent cytotoxicity (CDC). Furthermore, particular AQP4 isoforms can form supramolecular orthogonal arrays that arrange in a manner that benefits autoantibody multimeric complexes supporting Fc-Fc interactions that are critical for CDC (63). Whether or not a similar phenomenon may occur in the context of AChR-specific MG has yet to be explored. However, at the NMJ, the AChR is tightly clustered by the intracellular scaffolding protein, rapsyn; thus, such organized formations of self-antigen may support Fc-Fc interactions facilitating efficient CDC, by AChR autoantibodies recognizing particular epitopes. Thus, parallels to pathogenic mechanisms that occur in NMO warrant investigation.

## Immunopathology of MuSK Myasthenia Gravis

### Characterizations of B Cells in MuSK Myasthenia Gravis

Among the notable differences in the pathophysiology of MuSK and AChR MG is the role of the thymus in causing disease. As discussed previously, the thymus is a source for B cells specific for AChR in patients with AChR MG. While conspicuous in its pathogenic role in many AChR MG patients, abnormal thymus histopathology is not observed in patients with MuSK MG (64, 65). There are very few studies in which MuSK autoantibody-producing B cells have been identified and isolated. To date, these B cells have been found only in the circulation (66) and have a memory B cell or short-lived circulating plasmablast phenotype (19, 67). The variable region sequences of these autoantibodies revealed that they exhibit hallmarks of affinity maturation, including a high frequency of somatic hypermutation. They are oligoclonal, but the number of mAbs is currently too limited to draw firm conclusions about whether or not they share unique repertoire properties with each other, such as restricted variable region gene usage.

### Properties of MuSK Myasthenia Gravis Autoantibodies

MuSK autoantibodies differ from their AChR-specific counterparts in terms of their subclass. MuSK autoantibodies are predominantly of the IgG4 subclass (68). Antibodies of the IgG4 subclass have an ambivalent role in immunity. In the context of allergy, especially studied in beekeepers, IgG4 antibodies dampen inflammatory reactions by competing with disease-mediating IgE antibodies for the same antigen, namely phospholipase A2 (69–71). In addition, beekeepers who were tolerant to bee venom had increased venom-specific IgG4 levels, while the venom-specific IgE levels were almost undetectable (69). This change toward protective IgG4 production seems to be modulated by sensitized regulatory T and B cells that secrete IL-10, leading to increased IgG4 and decreased IgE levels (72–74). The protective function of IgG observed in allergies has also been described in the autoimmune disease systemic lupus erythematosus (SLE) (75); the amount of complement deposition was negatively correlated with the amount of IgG4 antibodies in an *in vitro* deposition assay. The mechanism underlying this observation might be that IgG4 antibodies block and prevent autoantibodies of other subclasses and their effector functions – a similar approach using an antibody lacking effector functions called aquaporumab is currently under development in the treatment of NMO (76). In contrast to this regulatory and anti-inflammatory aspect of IgG4 immunology, several autoimmune disorders including MuSK MG, pemphigus vulgaris (PV), and chronic inflammatory demyelinating polyneuropathy (CIDP), include pathogenic IgG4 autoantibodies (77–79). IgG4 antibody effector function is clearly distinct from that of IgG1 antibodies. IgG4 subclass antibodies cannot activate the complement cascade via the classical pathway due to their poor affinity for C1q and Fc receptors (80–82). Thus, similar to the observed protective functions of IgG4 antibodies, MuSK autoantibodies exert their pathogenicity through blocking the interaction between MuSK and LRP4 that is required for the clustering of the AChR (83, 84).

**FIGURE 1:**
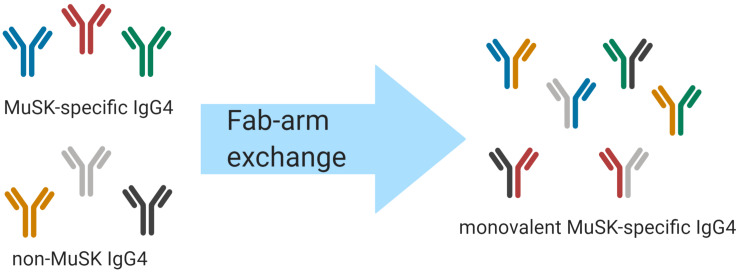
Schematic of IgG4 Fab-arm exchange in MuSK MG. Human IgG4 antibodies can participate in a process termed Fab-arm exchange. In the MuSK autoantibody subtype of MG, MuSK-specific IgG4 autoantibodies can undergo Fab-arm exchange with other circulating IgG4 antibodies. The antibodies that were formerly divalent – with two identical antigen binding sites – become monovalent and bispecific after a heavy and light chain pair is switched with a heavy and light chain pair of another antibody. The process is thought to be critical for the development of pathogenic autoantibodies in MuSK MG.

An intriguing feature of human IgG4 antibodies is their exclusive ability to participate in “Fab-arm exchange” (Figure 1) (85). Fab-arm exchange (FAE) is a process whereby antibodies can dissociate to produce two identical half molecules each formed of a heavy chain and a light chain (Figure 1). These half molecules can then recombine, producing antibodies with two distinct variable regions which cannot crosslink identical antigens and are therefore functionally monovalent. FAE has been shown to play an important role in the immunopathology of MuSK MG (84). The mechanism of FAE is not fully understood, but key amino acid residues and conditions required for the exchange process have been elucidated. The Fc region plays a key role in the process of FAE. Two major interactions between the Fc regions are crucial for holding the two parts of the IgG molecule together: interchain disulfide bonds in the core hinge region and non-covalent interactions in the third constant heavy domain (CH3) of the respective chains (85, 86). Specific amino acids at these sites in the IgG4 molecule facilitate the dissociation of the two halves of the antibody and enable FAE. Although the sequences of the different IgG subclasses share similarities, some small changes have a large effect on the stability of the IgG molecule. The core hinge region in IgG1 contains the motif 226cys-pro-pro-cys-pro230 which confers stability. By contrast, IgG4 contains a serine at position 228 which enhances hinge flexibility and promotes dissociation of the molecule (87). Site-directed mutagenesis studies have shown that replacing the endogenous serine with a proline at position 228 in IgG4 reduces the formation of half molecules (88, 89) and prevents FAE from occurring *in vivo* and *in vitro* (89–91). Moreover, non-covalent interactions in the third constant (CH3) domain play a vital role in holding the two chains together (87). The lysine at position 409 in the CH3 region of IgG1 contributes to the stability of the molecule. Mutating this lysine to an arginine residue has been shown to destabilize the interchain links and lead to its dissociation into half molecules (92–95). Mutating lysine to arginine may not appear to be a particularly significant substitution but it should be noted that there are there are other examples of this mutation having rather profound effects *in vivo* (96, 97). Producing an arginine-to-lysine mutation in IgG4 CH3 was shown to cause a 10- to 100-fold change in the dissociation constant, which was enough to make the difference between enabling and inhibiting FAE (86, 98). FAE can occur under certain physiological conditions. Under non-reducing conditions IgG4 acts as a regular bivalent antibody (99). IgG4 can participate in half molecule exchange only under reducing conditions, which can be induced *in vitro* with low concentrations of the reducing agent glutathione (GSH) (85, 100). It is thought that the reduction of interchain disulfide bonds in the hinge region is a pre-requisite step for FAE (87, 101). Moreover, the reaction occurs more efficiently at physiological temperatures rather than at room temperature (85). Other factors contributing to FAE alongside amino-acid sequence, temperature, and reducing environment have been considered (e.g., time course for exchange, IgG ratio, and concentration of antibody), however, these factors have not been explored in depth (86, 102, 103). It was also found that plasma components have only a minor effect on the extent and duration of FAE *in vitro* (103).

### Comparison Between MuSK Myasthenia Gravis, Pemphigus Vulgaris, and Chronic Inflammatory Demyelinating Polyneuropathy

MuSK MG shares several features with other autoimmune diseases like PV and CIDP. PV is an autoimmune disorder characterized by autoantibodies that target integral parts within the skin structure that are important for cell adhesion (104–108), most often desmoglein 1 and desmoglein 3 (109, 110). Consequently, PV manifests with skin blisters, often involving the oral mucosal membrane (111). CIDP patients present similarly to MG with muscle weakness. CIDP is a heterogeneous autoimmune disease affecting peripheral nerves. Autoantibodies in CIDP interrupt the conduction along the nerves (112), in contrast to MG where the immunopathology is located at the NMJ. CIDP autoantibodies have been found to target contactin 1, neurofascin, and other self-antigens, which are associated with the node of Ranvier (79, 113). The autoantibodies in PV and CIDP are predominantly of the IgG4 subclass; thus sharing a key feature with MuSK MG (77–79). However, the only disease in which FAE has, to date, been shown to play a role in is MuSK MG (84). Yet, evidence is available showing that FAE may be a common occurrence in human biology. The biological therapeutic natalizumab was engineered using the IgG4 subclass; it has a wildtype core hinge region that does not contain the stabilizing serine to proline mutation at position 228 (114). It has been shown that natalizumab exchanges Fab-arms with endogenous human IgG4 in natalizumab-treated individuals (114). However, the role of FAE in healthy individuals is currently not known.

FAE plays a key role in the pathogenicity of MuSK autoantibodies. Several passive transfer models have shown that the IgG4 autoantibodies in MuSK MG are pathogenic *in vivo* (115, 116) and that serum-derived IgG4 autoantibodies are also pathogenic after Fab-arm exchange using an established *in vitro* assay (117). Using monoclonal patient isolated autoantibodies, we and others recently found that divalent MuSK autoantibodies could slightly induce agrin-independent AChR clustering by crosslinking MuSK at the NMJ leading to autophosphorylation of MuSK (66, 67). A stronger pathogenic effect of isolated MuSK autoantibodies was observed by testing these as monovalent Fabs (thus emulating FAE products); the monovalent Fabs could not crosslink MuSK on the cell surface and exerted their pathology through blocking the MuSK and LRP4 interaction, leading to a robust reduction in AChR clusters (66, 115–118).

The effect of valency on pathogenic autoantibodies in CIDP is not known at this time. The pathogenic effect of monovalent Fabs has been demonstrated for PV (119–122), but pathogenic autoantibodies in PV can be divalent as well, indicating that pathogenicity is not dependent on FAE as it is in MuSK MG. That FAE appears to be necessary for efficient pathology in MuSK MG, but not PV, may be explained by the very different functional contributions each of the antigen targets make to the cells in which they are expressed. MuSK is a transmembrane kinase, responsible for delivering a signal to induce AChR clustering. Desmogleins are adhesion-molecule superfamily members that mitigate cell-to-cell interactions. Therefore, when MuSK is crosslinked by a divalent autoantibody, which induces phosphorylation, the resulting affect is agonistic (*signal delivered*), and that is coupled with the blocking of the interaction between LPR4 and MuSK. When the autoantibody is monovalent (after FAE), the only effect is blocking LPR4 interaction and consequential pathology due to failed signaling for AChR clustering. On the other hand, in PV, autoantibody binding to the desmogleins interrupts their binding to partners; thus, monovalent or divalent autoantibody binding equally effect interference. Overall, not enough mechanistic detail is available regarding the role of FAE in CIDP, PV, and most intriguingly, normal immune responses in healthy individuals.

Finally, similarities in the immunopathology of CIDP, PV, and MuSK MG are also observed in responses to treatment. Patients with all three diseases respond very well to the treatment with the B cell depleting drug rituximab (123–125), the benefit of which can last for years (126). This effect seems to be common among autoimmune diseases that are mediated by IgG4 and is further discussed below.

## LRP4 and Seronegative Myasthenia Gravis

Compared to what we know about AChR MG, there is a scarcity of information concerning the immunopathology of LRP4 MG and SNMG. However, that which is understood of the immune mechanisms contributing to LRP4 MG indicate that similarities to AChR MG can be found. Autoantibodies against LRP4 have been found in patients who were previously identified as seronegative (6, 127, 128). These autoantibodies were shown to disrupt the Agrin-LRP4 signaling and to be mainly of the complement activating IgG1 subclass (6). In contrast to findings, which show that that AChR and MuSK autoantibodies are specific for MG (129), LRP4 antibodies appear to cross disease boundaries. For example, LRP4 autoantibodies have been detected in some patients with amyotrophic lateral sclerosis (ALS) who presented with myasthenic symptoms (130, 131). The role of the thymus in LRP4 MG was recently investigated in a small pilot study (132). This study showed that there was a heterogeneity in thymus morphology among the four tested patients. Two out of these four patients seemed to benefit from thymectomy after a one-year follow up, while one of those two patients needed no additional treatment after thymectomy. Overall, there have been few investigations of the immunopathology that contributes to LPR4 MG, and thus caution should be taken such as not to generalize these early findings.

Some patients originally categorized as seronegative were later found to have detectable AChR, MuSK, or LRP4 autoantibodies due to either improved test sensitivity or increased titers above the lower limit reference on repeat measurement (133). Other SNMG patients remain defined by the absence of detectable autoantibodies. This could be the consequence of sensitivity limitations within our current detection assays or due to the fact that other unidentified autoantigens are present within these patients. Indeed, when highly sensitive cell-based assays (CBAs) were introduced for the detection of AChR autoantibodies, a number of SNMG cases tested positive for circulating AChR autoantibodies. Similarly, both MuSK MG and LRP4 MG were identified through investigating novel targets in SNMG patients. Several new autoantibody targets within the NMJ, including agrin, collagen Q, cortactin, and the voltage-gated potassium channel, Kv1.4, have been proposed (134–138); however, these autoantibodies have not, as yet, been shown to have pathogenic capacity. Autoantibodies against the intracellular proteins, titin, and the ryanodine receptor were found to be potential candidate biomarkers for disease monitoring in MG (139–141). These autoantibodies together with other striational autoantibodies have been observed in patients with MG (142), however, their direct contribution to pathogenicity is unlikely, given the intracellular location of their targets.

Some SNMG patients respond to immunosuppression, IVIG, and plasmaphereses, which indicates that an autoantibody-mediated mechanism may contribute to the pathology (143, 144). The effect of thymectomy in SNMG is not clear. Some studies found positive therapeutic effects similar to seropositive MG (145–147), while other studies failed to show a beneficial effect (144, 148). Optimal treatment paradigms and outcomes for SNMG patients are uncertain. Further compounding the problem, SNMG patients are often not included in clinical trials in which autoantibody-positive patients participate. Consequently, these patients are not managed with standardized treatment approaches due to a lack of understanding of the disease mechanisms. It is reasonable to suspect that SNMG is, in many cases, a misnomer. Rather SNMG is likely a heterogeneous disease consisting of patients who have pathogenic autoantibodies directed against indeterminate NMJ targets, or known autoantibodies (AChR, MuSK, or LRP4) that are below the level of detection with current commercial diagnostic tests.

## Therapeutic Intervention Further Highlights the Divergent Immunopathology of AChR and MuSK Myasthenia Gravis

The current standard of care for the treatment of MG largely targets MG symptoms rather than specific immune components underlying the disease subtypes. Immunosuppressive agents (i.e., corticosteroids) are used most often for this purpose (149). Cholinesterase inhibitors, such as pyridostigmine, prevent the degradation of acetylcholine and thus increase its availability at the NMJ, thus improving neuromuscular transmission (150–153). Some patients do not respond well to these therapies due to side effects or incomplete clinical benefit. Approaches that more directly target the immune system have recently shown promising effects. While such immune-modifying biologics in MG have proven to be therapeutically beneficial, they have also provided a unique opportunity to further understand MG immunopathology. Laboratory-based study of patient-derived material before and after such therapeutic interventions have been leveraged to provide immunomechanistic detail that would otherwise not be possible in laboratory-based translational investigations. These include B cell depletion, inhibition of complement, targeting the BAFF/APRIL system, resection of the thymus, and interruption of IgG recycling.

### Thymectomy

Thymectomy is a well-established treatment option in AChR MG. Even before experimental studies demonstrated the possibility that thymus resident B cells could produce AChR-specific autoantibodies, removal of the thymus had already been widely accepted as a treatment option for AChR MG (154, 155). Recently, thymectomy was formally confirmed to be beneficial in AChR MG patients compared to treatment with corticosteroids alone (156, 157). The thymus of around 70% of AChR MG patients is hyperplastic and populated by B and T cells, while the thymus of healthy subjects is involuted by adulthood (158, 159). Thymectomy was shown to generally lead to a reduction in overall AChR autoantibody titer, although AChR autoantibody titers almost never become undetectable (160, 161). In a subset of patients, only modest decreases of the AChR titer can be found (162). Whether patients with modest changes in AChR titer are less likely to go into remission is not known yet (160). About half (40–50%) of AChR patients experience long-term remission without relapse following thymectomy when followed for up to 20 years post-procedure (163, 164). While clinical improvement is observed in half the patients following thymectomy, complete remission is not achieved in many patients.

It is not clear why there is a heterogeneous response to thymectomy. Several retrospective studies explored factors associated with non-remission after thymectomy (162–164). Non-ocular MG (164), thymoma (162–164), specific surgical techniques (162, 163), duration of disease prior to resection (162), and age (163) have all been associated with a failure to respond to thymectomy. A series of studies have noted the failure of specific thymectomy approaches to remove the entire thymus, resulting in residual thymus tissue and symptoms (165, 166).

The thymus may be the site in which AChR B cells are initially activated and then mature. It is reasonable to speculate that autoantibody-producing B cell clones residing in the thymus can also populate compartments in the periphery. Consequently, surgical resection removes autoantibody-producing B cells but those which have emigrated from the thymus may continue to contribute to disease. The distribution of pathogenic AChR autoantibody-producing cells in anatomic compartments aside from the thymus, such as in the lymph nodes and bone marrow of patients with AChR MG (44, 45), must be considered. While thymectomy will remove a large fraction of thymus-resident autoreactive B cells and the cells that support their development, this treatment is performed only once the disease is established. This may be too late to halt disease progression, as those thymic B cells that have emigrated contribute to ongoing disease. Accordingly, combination therapies aimed at targeting residual thymus-related B cells may prove to be a valuable part of a potential therapeutic strategy. The use of B cell depleting agents such as rituximab – currently used for the treatment of MG – may fit this approach well. While no controlled studies have specifically investigated the effect of B cell depletion following thymectomy, there is some evidence (167) showing that patients who underwent thymectomy respond to rituximab similarly to those who did not have the surgery. Firm conclusions cannot be drawn from restricted numbers of patients, but these data may again point toward the key role of rituximab-resistant plasma cells in AChR autoantibody production. Accordingly, consideration may also be given to anti-CD19 based treatments that would additionally target plasma cells thought to be spared by rituximab (168).

While it is clear that thymectomy depletes a population of B cells that secrete AChR-specific autoantibodies, it is unclear if therapeutic benefit arises from the removal of pathogenic B cells alone. That thymectomy includes the removal of pathogenic T cells, including Tregs that are defective in suppressing T cell proliferation and conventional T cells that resist Treg-mediated suppression (169), supports the idea that non-B cell related disease mechanisms may be interrupted by the procedure. Abnormal thymus histopathology is not observed in patients with MuSK MG (64, 65). Given the positive effect of thymectomy on AChR MG, including cases without measurable thymic abnormalities (170), thymectomy has been applied as a treatment option for MuSK MG. However, thymectomy has not been demonstrated to improve clinical outcomes for MuSK MG patients (170, 171). Consequently, thymectomy is a possible therapy for AChR MG only – regardless of the presence or absence of thymic abnormalities.

### B Cell Targeting Therapies

#### Anti-CD20 and Anti-CD19 Antibodies

CD20 is a surface molecule that is expressed on B cells at almost every step of B cell differentiation. Only pro B, pre B I, and plasma cells do not express CD20 (172). The anti-CD20 mAb rituximab (RTX) is currently a treatment option for MuSK MG and has been trialed for the treatment of AChR MG. A case report on the successful treatment of AChR MG in a patient treated for non-Hodgkin’s lymphoma offered the first evidence in support of the use of B cell depletion therapy in MG (173). Several groups have subsequently investigated the efficacy of RTX in the treatment of AChR and MuSK MG (125, 174, 175). These studies have demonstrated 100% complete stable remission for the use of RTX in MuSK MG (125, 174), while 56% of AChR patients experienced a relapse within an average of 36 months after treatment – a finding that was replicated in another similar independent study (125, 167). Although MuSK MG patients respond very well to treatment with RTX, relapses do occur, and the relapse rate is dependent on the applied RTX treatment protocol (176). Consistent reductions in AChR autoantibody titers and clinical improvement were demonstrated in a cohort study involving six patients (174). These results were similar to those from an independent study demonstrating symptomatic improvement for AChR patients undergoing therapy; however, no corresponding fall in AChR autoantibody titer was observed (125).

In general, there is a poor correlation between the titer of AChR-specific autoantibodies and overall clinical progress (125). The titer of MuSK autoantibodies associates with the clinical improvement observed after B cell depletion therapy, the same is not true in AChR MG. These contrasting results are well highlighted by a study (125) that demonstrated clear clinical improvement in both AChR and MuSK MG, but only MuSK autoantibody titers diminished, while intra-patient AChR autoantibody titers increased, decreased, or stayed the same. The poor correlation with clinical severity has been known since the earliest initial studies that established the use of assays measuring AChR autoantibody titers (160). This likely reflects the polyclonal nature of anti-AChR antibodies, their different specificities, subclasses, local concentrations and complement, modulating, and blocking activity. It is important to point out that the assays used to diagnose MG by measuring AChR binding, provide no information whatsoever on their pathogenic capacity. It is possible that a fraction of autoantibodies that bind in the laboratory assays have little pathogenic capacity *in vivo*. Furthermore, circulating AChR autoantibodies, by definition, are not present at the site where the disease pathology occurs (the NMJ). Combinations of these factors may contribute to a disassociation between circulating titer and disease severity. However, several studies have shown that intra-patient longitudinal AChR autoantibody titers may correlate with disease severity progress (160, 177). Establishing the use of relative – as opposed to absolute – AChR autoantibody as a trial endpoint may prove to be a useful biomarker in the future. Given that the RIA or CBA used to diagnose patients and provide AChR titer values are wholly unable to discriminate between the detection of these autoantibodies and their pathogenic properties, applying an assay suite that can quantitate the extent of AChR autoantibody-mediated complement activation, blocking, or modulating within an individual patient may associate with disease severity better than simple AChR binding titer measurements.

Recently, a phase-2 trial called BeatMG, designed to test the efficacy of RTX treatment in AChR patients with mild to severe disease, ended (ClinicalTrials.gov Identifier: NCT02110706), while a phase-3 trial called RINOMAX is currently in progress with patients that have moderate to severe disease (ClinicalTrials.gov Identifier: NCT02950155). The BeatMG study showed slightly favorable effects of treatment with rituximab especially in patients with more severe courses of disease, although there was no statistical difference between the rituximab and placebo groups (178). The data from the clinical trials aimed at investigating the efficacy of RTX in MuSK and AChR MG offer unique insight into the distinct immunopathology of these two MG subtypes (125, 167, 174). In general, RTX does not efficiently deplete tissue-localized B cells in lymph nodes, tonsils, and bone marrow (179–182). Moreover, RTX has been particularly efficacious in diseases mediated by pathogenic IgG4 antibodies such as PV, and CIDP in addition to MuSK MG (124, 183). Long-lived plasma cells populations residing in the thymus produce some of the circulating AChR-specific autoantibodies; plasma cells express low levels of CD20 (32, 34). In contrast, circulating plasmablasts, such as those that secrete MuSK-specific autoantibodies, typically express higher levels of CD20 than their tissue resident plasma cell counterparts (19, 184), although some refractory B cell clones were found to emerge during relapse in MuSK MG after treatment with RTX (185). Thus, differences in the efficacy of RTX in AChR and MuSK MG may reflect differences in the tissue localization of disease-causing B cell subsets and/or the susceptibility of different autoantibody-producing B cell subsets (*plasmablasts in MuSK MG and plasma cells in AChR MG, each expressing different levels of CD20*) to anti-CD20 depletion.

Although it is well-understood that MuSK MG patients responds remarkably well to B cell depletion therapy, there are patients who respond less well to this treatment and a small fraction who do not improve (186). This highlights the heterogeneity that is invariably observed among MG patients. While it is not understood why non-responders have emerged, one can speculate that some MuSK patients may produce autoantibodies from a subset of B cells that do not express CD20, such as plasma cells as in AChR MG, or they utilize plasmablasts with low surface CD20 expression levels. Other possible mechanisms include diminished complement activity, the mechanism by which anti-CD20 mediates cell death.

Alternative strategies of B cell depletion therapy have recently emerged, including mAbs targeting CD19. CD19 is a gene surface marker that is expressed over a wider range of B cell subsets than CD20. CD19 is expressed before the expression of CD20 in pro-B cells and declines after the expression of CD20 in plasma cells. A larger proportion of plasma cells expresses CD19 in comparison to CD20 (187). Thus, anti-CD19 agents could potentially enhance the depletion of disease-causing plasma cell populations. MEDI-155 or inebilizumab is an IgG monoclonal antibody that was initially demonstrated to be more effective than anti-CD20 depletion in EAE, a mouse model for multiple sclerosis (188, 189). This study showed that the improved efficacy of anti-CD19 depletion could be explained by the depletion of plasma cells in the bone marrow. Consequently, two phase 1 trials were initiated for its use in relapsing-remitting multiple sclerosis (190) and systemic sclerosis (191) with promising results. Moreover, a phase-2/3 clinical trial was initiated for its use in the treatment of NMO called NM-omentum (192). The NM-omentum trial showed a clear efficacy of the treatment with anti-CD19 over placebo (168). The treatment with anti-CD19 therapy is a possible option for both MuSK and AChR MG. In comparison to anti-CD20 based therapy, targeting CD19 could have an increased effect on the AChR autoantibody-producing B cell subsets.

#### Proteasome Inhibitors

Given that plasma cells are suspected of playing an important role in the production of disease-causing autoantibodies in AChR MG, direct plasma cell depletion with proteasome inhibitors has been proposed for the treatment of this MG disease subtype. Bortezomib is such a proteasome inhibitor, and it was shown that it could directly deplete plasma cells (193–195). The proteasome is connected to cell homeostasis and promotes protein clearance of cell apoptosis associated and misfolded proteins (196). Bortezomib was demonstrated to be efficacious for the treatment of hematologic autoimmune diseases such as autoimmune hemolytic anemia (AIHA), immune thrombocytopenia (ITP), and thrombotic thrombocytopenic purpura (TTP) in a phase-2 trial (197). Bortezomib (198) and other proteasome inhibitors (199) were first shown to be beneficial in EAMG, a mouse model for MG. Moreover, *in vitro* studies of AChR MG patients showed that bortezomib can eliminate thymus-derived plasma cell populations, reducing pathogenic IgG as well as total IgG levels (194). Consequently, a phase-2 trial called TAVAB that investigated the use of bortezomib in generalized AChR MG, rheumatoid arthritis (RA), and SLE was initiated in 2014, although results are still not yet available (ClinicalTrials.gov Identifier: NCT02102594) (200). The plasmablasts that are thought to produce autoantibodies in MuSK MG are not targeted by proteasome inhibitors. Accordingly, proteasome inhibitors may be a possible treatment option for AChR MG only.

#### Targeting the BAFF/APRIL System

The survival of B-cells is regulated in part by the BAFF/APRIL system. This system consists of the two ligands B-cell activating factor (BAFF/BLyS/TALL-1) and a proliferation-inducing ligand (APRIL), and the three receptors, B-cell activating factor receptor (BAFF-R), B-cell maturation Ag (BCMA), and transmembrane activator and CAML interactor (TACI) (201). BAFF and APRIL are B cell stimulatory molecules that promote B cell proliferation, autoimmunity, somatic hypermutation and mediate B cell survival (202). The BAFF/APRIL system is a highly balanced system controlling B cell survival and proliferation. High levels of BAFF lead to an imbalance towards B cell proliferation. The occurrence of high levels of ligands and soluble receptors of the BAFF/APRIL system is associated with B cell pathologies (203–206) and high levels of BAFF are linked to autoimmunity (203, 207, 208). The anti-BAFF antibody belimumab was shown to be efficacious in the treatment of SLE in a phase-3 randomized controlled trial and it was approved by the FDA for the treatment of SLE (209). Elevated serum BAFF levels have been observed in both AChR MG and MuSK MG patients, and BAFF levels were shown to correlate with autoantibody titer (210–212). The results from a recent randomized controlled trial on the use of belimumab in AChR MG, however, failed to meet the primary endpoint of a change in quantitative myasthenia gravis (QMG) score (213). High serum BAFF levels have been shown to correlate with poor responses to rituximab in RA and Sjogren’s disease, raising the possibility that combination therapy with B cell depleting agents may hold promise (81, 214, 215). Several other approaches targeting the BAFF/APRIL system have been investigated, including atacicept (a soluble decoy receptor for BAFF and APRIL) which showed beneficial effects in SLE and RA (216–218). However, adverse effects in its use for the treatment of multiple sclerosis indicate that it may have a more complex role within the immune system (219). Additionally, γ-secretase inhibitors were recently described as an add-on therapy for multiple myeloma, as the inhibition of the shedding of BCMA was shown to work synergistically with CAR-T cell therapy (220). BAFF-R, BCMA, and TACI are expressed differently during all steps of B cell development (172). Consequently, targeting the BAFF/APRIL system is a potential therapeutic avenue for both MuSK and AChR MG.

### Complement Inhibitors

AChR MG autoantibodies are mainly of the complement-inducing IgG1 subclass. Accordingly, the complement system has been shown to be an effective target for the treatment of AChR MG. Two different therapies are available and have been tested in refractory AChR positive generalized MG. The first, eculizumab, is a humanized mAb that binds to C5 and thus inhibits the terminal complement pathway (221). Eculizumab showed positive effects in paroxysmal nocturnal hemoglobinuria (PNH) (222, 223) and was shown to be beneficial in atypical hemolytic uremic syndrome (aHUS) (224–226). Additionally, eculizumab was successfully tested in a clinical trial for the treatment of NMO with a primary endpoint of total relapse frequency (PREVENT Study; ClinicalTrials.gov Identifier: NCT01997229) (227, 228). After a promising pilot phase-2 trial of eculizumab in AChR positive generalized MG (229, 230), a phase-3 clinical trial of eculizumab was initiated (REGAIN; ClinicalTrials.gov Identifier: NCT01997229) (231, 232). Although the study did not achieve its primary endpoint of a statistical difference in the Myasthenia Gravis-specific Activities of Daily Living scale (MG-ADL) score for patients, additional sensitivity analyses of different MG-related scores including the MG-ADL showed improvements in the eculizumab group in comparison to the placebo group. Therefore, eculizumab was approved for the treatment of generalized AChR MG. Pointing again to heterogeneity within MG patient subtypes, it was interesting to observe that in the phase-3 clinical trial, 40% of AChR autoantibody-positive patients did not meet the trial endpoint. Furthermore, it is now appreciated that some patients have a conspicuous and rapid response to eculizumab, while others do not respond or have a more protracted improvement. These results may reflect heterogeneity among patients in terms of the relative fractions of AChR autoantibody-mediated complement activation, blocking or modulating functions (discussed above).

Zilucoplan – a small molecule (synthetic macrocyclic peptide) – binds to C5 and inhibits the terminal complement pathway (233). A phase-2 trial showed significant improvement in generalized MG patients, leading to the approval of a phase-3 clinical trial which is currently in progress (RAISE; ClinicalTrials.gov Identifier: NCT04115293). In contrast to AChR MG, MuSK MG autoantibodies are mainly of the IgG4 subclass, which does not activate complement (discussed above). Thus, treatment with complement inhibitors, at this time, is likely to be mostly beneficial for AChR MG patients. Treatment of patients with SNMG or LRP4 MG with complement inhibitors could provide highly valuable information regarding mechanisms of immunopathology. In SNMG, beneficial outcomes would point toward autoantibody-mediated pathology, thus providing key insight toward understanding this disease subset. In LRP4 MG such outcomes would further support the role of complement activating autoantibodies in disease pathology.

### FcRn Inhibitors

Human IgG is present at high concentration in serum (approximately 7–17 mg/mL). The half-life of circulating human IgG is between 3 and 4 weeks. This high-circulating level and long half-life are not exclusively dependent on synthesis, but rather due to continuous salvage and recycling. The IgG recycling pathway is mediated by the neonatal Fc receptor (FcRn) (234). FcRn inhibitors, which block the interaction of FcRn with IgGs, effect degradation and fast clearance of IgGs and are leveraged as such as therapeutics for IgG-mediated diseases (235). An early stage trial of one FcRn inhibitor called efgartigimod in patients with AChR MG showed a reduction in the titer of pathogenic autoantibodies that was associated with an improvement in disease severity (ClinicalTrials.gov NCT02965573, EudraCT 2016-002938-73) (236). These findings suggest that FcRn inhibitors may be a valuable treatment approach for MG. Although FcRn inhibitors have not yet been formally tested in MuSK MG, they reduce the circulating levels of all IgG subclasses (including IgG4) (237). Consequently, this treatment modality has the potential to be effective in treating MuSK MG as well as AChR MG. Again, this treatment paradigm could be leveraged to provide highly valuable information regarding autoantibody-mediated mechanisms of immunopathology of LRP4 MG and especially SNMG as discussed above.

## Conclusion

**FIGURE 2:**
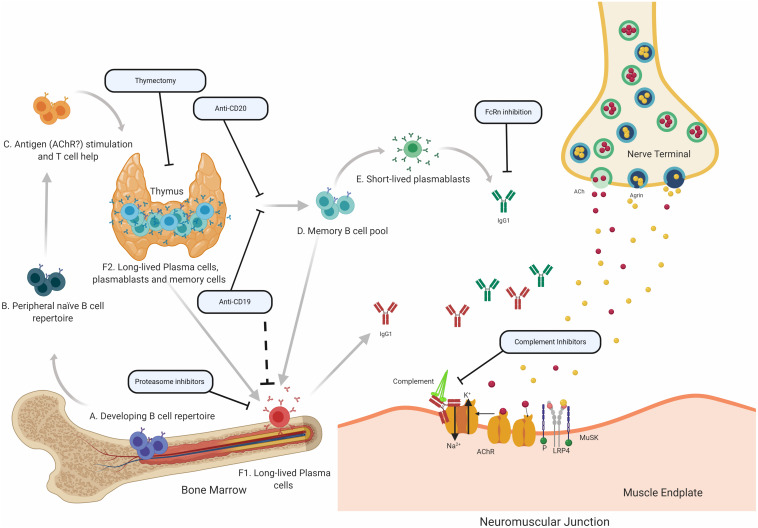
Speculative mechanisms of AChR MG immunopathology. The proposed mechanistic path to autoantibody production in AChR MG begins with naïve B cells (Steps A and B), which likely encounter self-antigen(s) and receive T cell help in the thymus (C). They can then differentiate into autoantibody specific memory B cells (D), which can be activated into antibody-secreting short-lived plasmablasts (E) or antibody-secreting long-lived plasma cells (F), which reside in the bone marrow (F1) and may also be present in the thymus (F2) of some patients with AChR MG. It is thought that long-lived plasma cells in the bone marrow and thymus make major contributions to AChR autoantibody production. Autoantibodies migrate to the NMJ where they bind to the AChR hindering the neuromuscular transmission by directly interrupting acetylcholine signaling at the AChR. Most of the antibodies are of the IgG1 subclass which can induce the complement cascade. Several therapeutic strategies target different parts of this process in AChR MG. Thymectomy is thought to directly remove autoantibody-producing B cell and other pathogenic cell subsets. B cell depletion, mediated by anti-CD20 antibodies is thought to remove autoreactive B cells, which includes memory cells and a subset of plasmablasts. Anti-CD19 antibodies can additionally target further subsets of plasmablasts and subsets of plasma cells. Proteasome inhibitors target plasma cells and may target the disease-causing long-lived plasma cells more efficiently. FcRn inhibitors increase the elimination of circulating IgGs, which is expected to also reduce the levels of pathogenic autoantibodies. Inhibiting complement activity, with complement inhibitors, results in the disruption of the pathogenic effector functions mediated by AChR autoantibodies.

**FIGURE 3:**
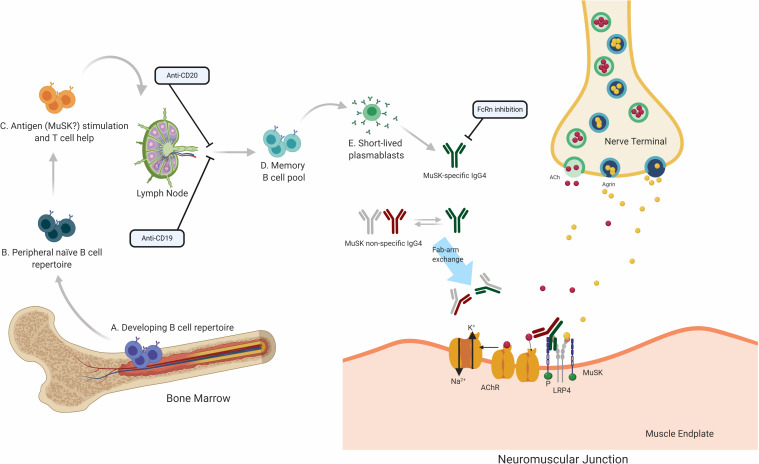
Speculative mechanisms of MuSK MG immunopathology. The proposed mechanistic path to autoantibody production in MuSK MG begins with naïve B cells (Steps A and B), which likely encounter self-antigen(s) and receive T cell help in the lymphoid tissue (C). They then differentiate into memory B cells (D) and antibody-secreting plasmablasts (E). Most autoantibodies in MuSK MG are of the IgG4 subclass. Antibodies of the IgG4 subclass can undergo the process of Fab-arm exchange with other antibodies of the IgG4 subclass. Consequently, the divalent mono-specific MuSK autoantibodies become monovalent bispecific autoantibodies. These autoantibodies migrate to the neuromuscular junction where they bind to MuSK hindering the neuromuscular transmission by blocking the LRP4 and MuSK pathway which is important for the clustering of the AChR. Several therapeutic strategies target different parts of this process in MuSK MG. B cell depletion by anti-CD20 antibodies is thought to remove B cells expressing CD20 which includes memory cells and a subset of plasmablasts. Anti CD19 antibodies can additionally target further subsets of plasmablasts. FcRn inhibitors increase the elimination of circulating IgGs, which is expected to also reduce the levels of pathogenic autoantibodies.

Translational laboratory-based research and clinical trials have both provided considerable evidence supporting the idea that the immunopathology of AChR and MuSK MG is distinct (summary in Table 1). In general terms, AChR MG is characterized by a key role for the thymus in its immunopathology and by autoantibodies of the complement activating IgG1 subclass, which are produced by plasma cells residing in the bone marrow, thymus, and other tissues (Figure 2). By comparison, MuSK MG autoantibodies are mainly of the IgG4 subclass, which undergo Fab-arm exchange as a prerequisite for pathogenic capacity. MuSK MG autoantibodies are thought to be produced by circulating short-lived plasmablasts (Figure 3). An understanding of these differences is valuable for defining different mechanisms that underlie human autoimmune disease. They are also highly important in considering treatment options, since an understanding of the immunopathology can inform such decisions. Within both the AChR and MuSK subtypes, further heterogeneity in disease course and wide-ranging response to treatment have both been observed. Furthermore, the immunomechanisms underlying SNMG and LRP4 MG still need to be more thoroughly understood. Accordingly, additional studies directed toward understanding the immunopathology, which associates with MG subtypes and the heterogeneity within each subtype, are needed. In such efforts, it is critically important that clinical trial leadership and laboratory-based translational research groups form partnerships so that highly valuable specimens, which provide deep insight into mechanisms, are properly curated and investigated.

**TABLE 1:** Autoimmune characteristics differentiating AChR and MuSK MG subsets and consequent response to immunomodulating therapies.

	AChR MG subtype	MuSK MG subtype
**Immunomechanisms**		
Thymus	Hyperplasia (in a subset)	Normal
Autoantibody IgG subclass	IgG1 and IgG3	IgG4
Role for complement	Major	Not significant
B cell subtype responsible for autoantibody production	CD20^neg^ Plasma cells	Plasmablasts
**Treatment response**		
Thymectomy	Clinical benefit	Clinical benefit not observed
Complement inhibitors	Clinical benefit	Clinical benefit not expected
Anti-CD20 (rituximab)	Some clinical benefit	Clinical benefit observed
FcRn inhibition	Clinical benefit expected	Clinical benefit expected
Anti-CD19	Clinical benefit expected	Clinical benefit expected
Proteasome inhibitors	Clinical benefit expected	Clinical benefit not expected

## Author Contributions

All authors listed have made a substantial, direct and intellectual contribution to the work, and approved it for publication.

## Conflict of Interest

KO’C has received research support from Ra Pharma and is a consultant and equity shareholder of Cabaletta Bio. KO’C is the recipient of a sponsored research subaward from the University of Pennsylvania, the primary financial sponsor of which is Cabaletta Bio. MF has received research support from Grifols. RN has received research support from the Alexion Pharmaceuticals, Genentech, Grifols, and Ra Pharma. The remaining authors declare that the research was conducted in the absence of any commercial or financial relationships that could be construed as a potential conflict of interest. The reviewer AC declared a shared affiliation, with no collaboration, with one of the authors, AB, to the handling Editor at the time of the review.
